# Tuning the Adhesive Properties of Soy Protein Wood Adhesives with Different Coadjutant Polymers, Nanocellulose and Lignin

**DOI:** 10.3390/polym13121972

**Published:** 2021-06-15

**Authors:** Milan Podlena, Martin Böhm, Daniel Saloni, Guillermo Velarde, Carlos Salas

**Affiliations:** 1 Department of Materials Engineering and Chemistry, Czech Technical University in Prague, Thákurova 7, 166 29 Prague, Czech Republic; m.podlena@seznam.cz; 2 Department of Forest Biomaterials, NC State University, 2820 Faucette Dr, Raleigh, NC 27607, USA; desaloni@ncsu.edu (D.S.); gjvelard@ncsu.edu (G.V.); clsalasa@ncsu.edu (C.S.)

**Keywords:** wood adhesive, biobased adhesive, soy protein, soy flour, lignin, shear strength

## Abstract

Commercial wood adhesives are based on products that contain formaldehyde; however, environmental and health concerns about formaldehyde emissions from wood products have influenced research and development efforts in order to find alternative, formaldehyde-free products for wood adhesives. In this work, different soy protein-based wood adhesives are proposed, and their performance is compared to commercial urea formaldehyde (UF) adhesive. Soy protein-based wood adhesives were prepared using either soy protein isolate (SPI) or soy protein flour (SF) with different coadjutant polymers: polyethylene oxide (PEO), hydroxypropyl methylcellulose (HPMC), cellulose nanofibrils (CNF) or polyvinyl alcohol (PVA) with and without addition of kraft lignin. The effects of the type of soy protein, solids content, coadjutant polymer and lignin addition were investigated. The wood adhesive formulations were tested on the bonding of hardwood (white maple) and softwood (southern yellow pine) and the dry shear strength of test specimens was measured according to method ASTM D905-08. The adhesive formulations with SPI achieved significantly higher values than those with SF. The dry shear strength of the adhesives varies depending on the coadjutant polymer, the wood species and the addition of lignin.

## 1. Introduction

Wood adhesives represent a large portion of the global adhesives market; for example, in 2018 the global wood adhesives market size was valued at USD 4.60 billion [1]. Wood adhesives are used in products such as plywood, particleboard, oriented strand board and medium density fiberboard, which are used in wood furniture and the construction industry. Most commercial wood adhesives are based on products that contain formaldehyde, namely urea-formaldehyde, melamine-formaldehyde, phenol-formaldehyde and resorcinol-formaldehyde [2,3]. However, environmental and health concerns regarding formaldehyde emissions from wood products have pushed towards reduction and regulation of the maximum allowable limits of such emissions in a given product. For example, in the U.S., the Environmental Protection Agency (EPA) released in 2016 the Formaldehyde Emission Standards for Composite Wood Products, intended to reduce formaldehyde emissions from different wood products including but not limited to hardwood plywood, medium-density fiberboard, particleboard and/or finished products containing these composite wood materials [4].

Considering the large size of the wood adhesive market and the current need for formaldehyde-free adhesives, there is opportunity for the development of alternative products that can help to achieve this goal. However, among the challenges in alleviating this problem is the development of wood adhesives that are not only formaldehyde-free, but that can also perform similarly well as those containing formaldehyde. This includes the tuning of the material adhesive properties [5]. Among the different materials studied, natural materials are at the front line because they offer multiple advantages such as availability from abundant biomass and agricultural waste, biodegradability and functionality. Starch and soy proteins are among the natural materials that have been studied for wood adhesives [2,3]. Soy protein adhesives have been extensively studied for their natural biobased potential [6,7,8].

Soybeans have high protein content, with main proteins composed mostly of glycinin and β-conglycinin, also called storage proteins. Different soy protein products are obtained and are commercially available after processing of soybeans to extract the oil. These products include soy protein isolates (SPI), soy protein concentrates (SPC) and soy flour (SF), with protein content of ca. 90%, 65% and 50%, respectively [9,10,11]. Soy flour is inexpensive because it does not require additional processing costs for removing carbohydrates. For this reason, it has been considered as a potential adhesive for the wood industry [7,10,11]. However, soy flour paste provides poor bonding properties by itself [12,13,14].

In the case of soybean proteins, the adhesive bond strength is highly influenced by the ability of the proteins to disperse well in water, and the interactions of the different amino acids in the proteins with the wood surface [15].

Proteins are sensitive to pH, temperature, added denaturants or salts [13]. A strong alkali treatment is required to achieve a strong bond line [16,17] for dispersion of soy protein. Good solubility of soy globulins is achieved at pH above or below the isoelectric pH (4–5) [9].

Other variables that influence the adhesive performance are the bonding conditions such as pressure and temperature. The hot and cold pressing can be used for curing soy protein adhesives [18]. Thermal analysis of unmodified soy isolate indicated thermal transition temperatures for soybean storage proteins of 73.8 °C (β-conglycinin) and 88.5 °C (glycinin) [19]. The viscosity of soy protein adhesives increases rapidly with increasing temperature up to 80 °C; afterwards it decreases [20]. As the temperature increases, the coalescence of protein globules is initiated and the proteins are rearranged, thereby causing an increase in the bond strength [20,21,22]. Nonetheless, the longer heating time (more than 1 h) can cause structural damages to the protein molecules and result in decrease of adhesive strength as has been investigated for adhesives modified with trypsin [18].

In the case of use as wood adhesives for applications with frequent moisture exposure, protein adhesives need to be chemically modified to improve their water resistance and shear strength [19,23]. The same effect is achieved by adding phenol resin [24] or low-cost lignin-based resin [25,26]. Lignin has also been evaluated as a phenol substitute in phenol-based adhesives [7,27]. Due to the economically complicated isolation of native lignin, soda lignin [28,29], organosolv lignin [30,31,32], lignosulfate [33,34,35,36] or kraft lignin [26] are dominantly used for industrial purposes [37]. In a recent report [38], Xin investigated how lignin modification that was prepared with SPI can improve the shear strength of bond line.

As mentioned before, one of the challenges that have been difficult to overcome is the performance of soy protein wood adhesives in conditions of frequent exposure to high moisture. To tackle this problem, different approaches have been proposed, including the crosslinking of the proteins with different chemicals, blending of soy protein adhesives with phenol formaldehyde glue and the use of enzyme-modified soy flour on the adhesive formulations. Some soybean-based commercial adhesives include the use of crosslinking resins such as cationic polyamidoamine-epichlorohydrin (PAE) resins [39]. The blending of soy protein with other synthetic materials and other proteins has been reported to produce adhesives with better performance than current adhesives; some cases include blends with blood, casein, phenol formaldehyde (PF) and phenol-resorcinol-formaldehyde (PRF), polyvinyl alcohol and polyvinyl acetate [6]. However, some of the proposed approaches still use formaldehyde or similar chemicals to crosslink the proteins.

In the case of wood adhesives for indoor applications, i.e., applications with less exposure to the elements, one of the alternatives that have been less explored is to modify the adhesive properties of soybean proteins by blending with other polymers. For instance, polysaccharides such as starch and cellulose derivatives, which are typically used as viscosifiers in many different applications, could be advantageous in adhesive formulations combined with soy proteins [10]. Of interest also are lignin [3,40,41] and nanocellulose materials [42]; the latter can act as a filler on the adhesive formulation and therefore enhance the adhesive performance.

In this work, different coadjutant polymers are evaluated in blends with soy proteins (soy flour and soy isolate) to produce adhesive formulations with and without lignin. The polymers include polyethylene oxide (PEO), hydroxypropyl methylcellulose (HPMC), cellulose nanofibrils (CNF) and polyvinyl alcohol (PVA).

Among the selected coadjutant polymers, hydroxypropyl methylcellulose (HPMC) is utilized as a rheology modifier in industrial applications, as an additive for tile adhesives in the construction industry, in cosmetics and food applications. HPMC has shown good film formation properties and binding properties in food-related applications [43].

Polyethylene oxide has shown synergistic effects with soy proteins as precursor solutions used to produce nanofibers via electrospinning [44,45] and solid films [46,47]. The mechanism of interaction of PEO with protein molecules has been suggested to be hydrogen bonding, hydrophobic and ionic interactions [48]. Similarly, polyvinyl alcohol has been blended with soy protein to produce fibers [49] and films [50,51]. Polyvinyl alcohol and polyethylene oxide were also found to be compatible with lignin in blends to produce electrospun nanofibers [52,53,54].

Nanocellulose has been the subject of research interest because of its interesting properties, namely high surface area, high aspect ratio, rheological properties and its eco-friendly nature. The utilization of nanocellulose in wood adhesive applications has also been studied. Melamine-urea-formaldehyde and urea formaldehyde wood adhesive formulations containing cellulose nanofibrils (CNF) in amounts of 1 and 3 wt% were used in the preparation of lab-scale particleboard and oriented strandboard [55]. The results showed an enhancement of the mechanical properties of both products with the addition of 1 wt% CNF. For instance, the boards prepared with urea formaldehyde exhibited up to a 10% increase in internal bond whereas the oriented strandboard showed a 16% enhancement in mechanical properties compared to formulations without CNF. It was suggested that the addition of CNF improved the fracture toughness and fracture energy of the boards [55]. Similar results were obtained when microfibrillated cellulose (MFC) was added as reinforcement phase of urea formaldehyde adhesives. The addition of up to 3 wt% of MFC increased the tensile shear strength by 6% compared to formulations without MFC. It was also observed that higher loads of MFC up to 5 wt% did not enhance but rather reduced the mechanical properties of the boards [56].

A previously published report indicates that the addition of microfibrillated cellulose to urea formaldehyde adhesives increased size of the adhesive particles and had a retarding effect on the curing process. Microscopy studies also showed that a larger part of the wood particles was covered with adhesive [57].

Other formaldehyde-free adhesive formulations have been proposed by using tannin-based resins [58,59,60,61]. Tannins are polyphenolic compounds present in wood. A recent work reports on the use of tannin-based resins with cellulose nanofibers. The addition of up to 2 wt% cellulose nanofibers improved both the viscosity and the internal bonding strength of the particleboards [62]. Soy-based, tannin-modified adhesives were also successfully used to bond plywood [63].

A number of recent studies are concerned with increasing the water resistance of soy protein-based adhesives [64,65,66,67,68]. However, improving the moisture resistance of the adhesive is not the purpose of this study.

It is the aim of this work to evaluate the synergy between soy bean proteins and different coadjutant polymers in order to produce formaldehyde-free adhesive formulations for potential application in wood composites. We hypothesize that because of the expected different molecular interactions between soy proteins and coadjutant polymers, synergistic effects will occur and favor the improvement in adhesive properties. Adhesive formulations containing either soy protein isolate or soy flour with different coadjutant polymers (polyethylene oxide (PEO), hydroxypropyl methylcellulose (HPMC), cellulose nanofibrils (CNF) and polyvinyl alcohol (PVA)) were prepared with and without lignin, and their adhesive properties (shear strength properties of the adhesive bond) were compared against commercial urea formaldehyde adhesive.

## 2. Materials and Methods

### 2.1. Materials

Sodium hydroxide (NaOH) solution (0.1 N (N/10)/Certified) titrated at 25 °C to pH 8.6 (Fisher Scientific, Waltham, MA, USA) with 10 vol% acetonitrile anhydrous (CH_3_CN), 99.8% (Sigma-Aldrich, St. Louis, MO, USA) was used as a liquid solvent. Soy protein isolate SPI Pro-Fam^®^ 974 (min. 90% protein content) and soy flour (52% protein content) were obtained as a gift by Archer Daniels Midland, Chicago, IL, USA, and were used as received. Polyethylene oxide (Average M_v_ 400,000) and hydroxypropyl methylcellulose (M_n_ ~ 86,000), polyvinyl alcohol, PVA (degree of hydrolysis of 98%, molecular weight of 125 kDa, trade name Mowiol 20–98) and kraft lignin with low sulfonate content were purchased from Sigma-Aldrich (St. Louis, MO). Cellulose nanofibrils (CNF) were prepared by high shear fibrillation of wood fibers as described elsewhere [69,70,71,72]. The composition of the chemicals was obtained from the technical datasheets and all chemicals were used as received.

The shear strengths of soy-based adhesives were compared to a commercial urea formaldehyde adhesive (UF). In this case, the Plastic Resin Glue—DAP Weldwood (DAPProducts, Baltimore, MD, USA) was used. 

Wood blocks of dimensions of 2” × 2” × ¾” (50.8 mm × 50.8 mm × 19 mm) were used as testing specimens. The white maple (*Acer saccharinum*) and southern yellow pine (*Pinus taeda*) were chosen as representative hardwood and softwood species.

### 2.2. Preparation of Protein Adhesives

The solvent was prepared by mixing dilute NaOH (0.1 N) with acetonitrile at the volumetric ratio of 9:1. The mixture was stirred for 10 min using a magnetic stirrer in a volumetric glass flask. The adhesive formulations were prepared at laboratory conditions, which were a temperature of 70 °F (21 °C) and 50% of the relative humidity. The conditions were monitored using hygro-thermometer.

For each of the formulations, the preparation was as follows: the appropriate amount of each solid in the adhesive formulation was slowly added to the solvent, which was kept being continuously stirred using a turbine stirrer LT400 (Yamato Scientific, Tokyo, Japan). The solids were added in the following order: coadjutant polymer first, protein powder second and lignin (when required) last. Each of the solids was added slowly and the stirring was continuous until full dispersion/solubilization was achieved before adding the next solid, typically 30–40 min per each solid.

**Figure 1:**
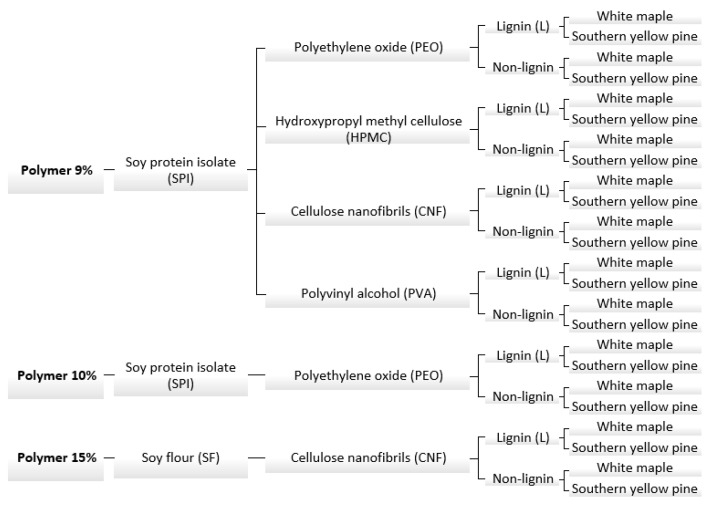
Experimental design diagram.

The experimental design can be seen in Figure 1; the formulations were composed of protein and different coadjutant polymers, namely polyethylene oxide (PEO), hydroxypropyl methylcellulose (HPMC), cellulose nanofibrils (CNF) or polyvinyl alcohol (PVA), each for a given formulation. The total concentration of solids in the formulations was kept at 9%, 10% and 15%, intended to evaluate the effect of solids content on rheology and adhesive properties of the formulation. The weight ratio of each of the solid components was kept as follows: 1 part of coadjutant polymer, 7 parts of protein and 2 parts of lignin (when required); when lignin was not added, the coadjutant polymer was kept the same and the balance was the protein content. The formulations were prepared with or without kraft lignin as described above. When the formulations achieved homogenous dispersion, the samples were stored for further use on the rheology and adhesive tests.

### 2.3. Basic Properties

Shear strength experiments were conducted according to method ASTM D905-08 (2021) [73]. The moisture content of wood specimens was stabilized at 50% relative humidity and 70 °F (21 °C). The initial moisture content of wood specimens was determined according to ASTM D4442-20 (2020) [74] and the density of dry wood in accordance with ASTM D2395-17 (2017) [75].

### 2.4. Preparation of Testing Specimens

**Figure 2:**
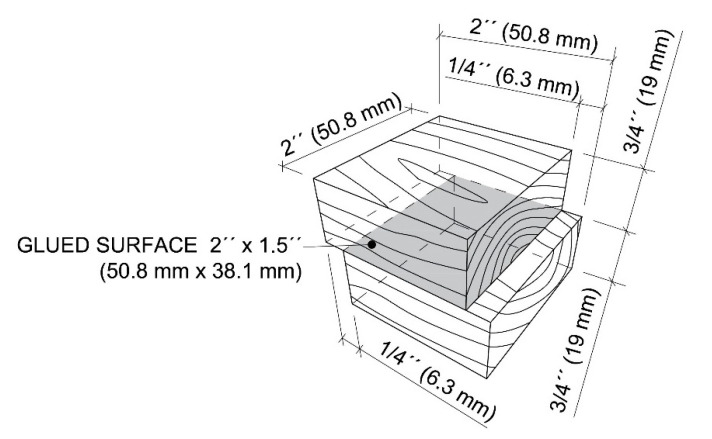
Dimensions of test specimens for testing of shear strength.

The standard testing method ASTM D905-08 was used to determine the shear strength between pairs of bonded wood blocks. A hardwood species (white maple) and a softwood species (southern yellow pine) were chosen. Wood specimens were selected with straight grain and without any significant defects such as knots, decay and discoloration. Firstly, the surfaces were smoothed using a planer S-290 (Baxter D. Whitney & Son, Greensboro, NC, USA) to the thickness of ¾ inches (19 mm). Afterwards, a straight-line rip saw SL-52 (Diehl Woodworking Machine, Wabash, IN, USA) and miter saw DWS780 (Dewalt Industrial Tool, Baltimore, MD, USA) were used to give 2 by 2 inches (50.8 mm by 50.8 mm) dimensions to the wood blocks. Figure 2 shows the standard form and dimensions of the test specimen. A total of 10 testing specimens were required for each type of adhesives. The adhesives were applied at a weight of 0.4 g, one-sided using a plastic pipette and spread on the bonded surface. The amount was checked by a balance APX-6001 (Denver Instrument, Bohemia, NY, USA). Once the adhesives were applied (within 4 min), blocks were assembled and taken to pressing immediately. The laboratory press Carver 3946 (1DI0A00, 12-ton capacity; Carver, Wabash, IN, USA) was preheated to a temperature of 340 °F (171 °C) and test specimens were pressed using 350 psi (2.4 MPa). After 10 min of pressing time, the specimens were conditioned for 24 h and prepared for mechanical testing. The environmental conditions were the same as at the chemical laboratory.

### 2.5. Preparation of Control Adhesive: Commercial Urea Formaldehyde Adhesive

The commercial UF adhesive Plastic Resin Glue—DAP Weldwood was purchased in a hardware store and used as indicated by the manufacturer. It is a powdered, precatalyzed adhesive with the following composition: urea-formaldehyde polymer, barium sulfate, tri calcium phosphate, ammonium sulfate, formaldehyde; specific gravity 0.7; thick liquid when mixed. The powdered adhesive was mixed with water at a weight ratio of 5: 3 (powder: water) according to the manufacturer technical datasheet. A testing series of 10 test specimens was prepared with southern yellow pine and white maple. The applying procedure, layer, environmental conditions were the same used for the soy protein adhesive formulations. Both platens of the press were heated to 140 °F (60 °C) and test specimens were compressed for 30 min using 300 psi (2.1 MPa) in accordance with the technical datasheet of the adhesive.

### 2.6. Moisture and Density of Wood Specimens

The moisture content of wood species was determined according to the standard ADTM D4442-20 by using the oven-drying method. The temperature of laboratory oven LBB2-18-1 (Despatch Industries, Mineapolis, MN, USA) was set to a temperature of 217 ± 36 °F (103 ± 2 °C). The endpoint was achieved when mass loss in a 3 h interval was equal or less than twice the selected balance sensitivity. At that point, the samples were weighted using the analytical balance PR2003 (Mettler Toledo International, Greifensee, Switzerland) again, and moisture content (*MC*) at the time of bonding was calculated according to Equation (1).

(1) $$MC\;\left(\%\right)=\frac{m_{w}-m_{0}}{m_{0}}\times 100$$

where *m_w_* is the initial mass of specimens (g) and *m*_0_ is the dry mass (g).

The oven-dry density (*ρ*_0_) was calculated in accordance with the standard test methods ASTM D2395-17 by using Equation (2).

(2) $$\rho_{0}\;\left(\frac{g}{cm^{3}}\right)=\frac{m_{0}}{V_{0}}$$

where *V*_0_ represents the oven-dry volume of the specimen.

The width, thickness and length of the oven-dry specimens were measured using a caliper 500-196-20 (Mitutoyo Corporation, Kawasaki, Kanagawa, Japan), and the volume was calculated.

### 2.7. Shear Strength Evaluation

**Figure 3:**
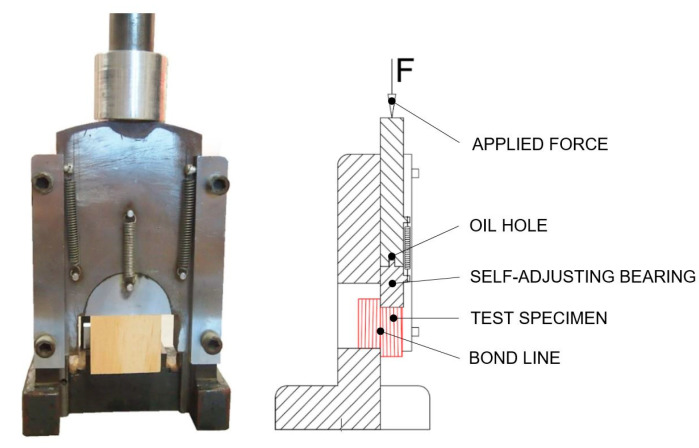
Test specimen set-up in self-adjusting shearing tool.

The shear strength of the adhesive bonds was measured by a mechanical testing machine Alliance RF/300 (MTS Systems Corporation, Eden Prairie, MN, USA). The shearing tool (Figure 3) was fitted to a testing machine and compression load was applied to test specimens. The grain direction was parallel to the direction of loading during test. The speed of the moveable crosshead was set up to 0.2 in/min (5 mm/min) constant speed. The software Testworks 4 (Testworks, USA) was used to analyze the strength at break points *σ* (*psi*) according to Equation (3). Testworks calculated breakpoints σ (pounds per square inch, psi) as a proportion of the measured peak loads *F* (*lbf*) and the bonded surface area *A* (*in*^2^) that were measured by a caliper 500-196-20 (Mitutoyo Corporation, Kawasaki, Kanagawa, Japan) with the precision for each sample.

(3) $$\sigma \;\left(psi\right)=\frac{F\;\left(lbf\right)}{A\;\left(in^{2}\right)\;}$$

The obtained results were evaluated using Statistica (TIBCO Software, Version 13.5, Palo Alto, CA, USA) software. A single-factor analysis of variance (ANOVA), factorial ANOVA and multiple comparison tests (post hoc: Tukey’s honestly significant difference (HSD) test and Tukey’s honestly significant difference for unequal sample sizes (unequal N HSD) test) were used for statistical evaluation of the measured data. All tests were performed at a significance of α = 0.05.

## 3. Results and Discussion

### 3.1. Rheology of Adhesive Formulations

**Figure 4:**
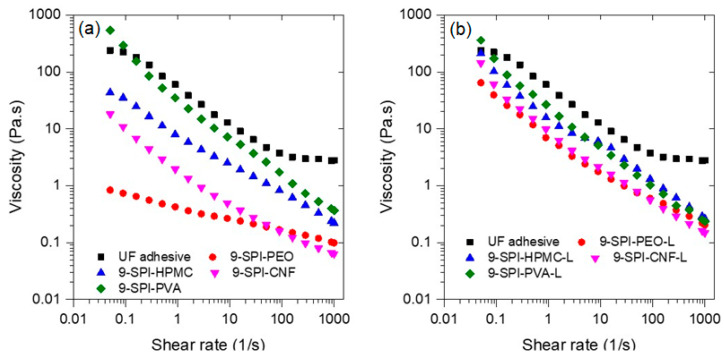
Flow rheology of studied adhesive soy protein isolate formulations (**a**) without and (**b**) with lignin.

The rheology of wood adhesives is an important variable to consider during application; a formulation with low viscosity will be less likely to remain on the surface of the substrate during application, whereas one with high viscosity will be difficult to spread on the surface. The rheology of the formulations with and without lignin compared to the UF commercial adhesive are shown in Figure 4. The results indicate that all the formulations exhibit shear thinning behavior; it can also be seen that the viscosity of adhesive formulations depends on the coadjutant polymer used and follows the trend SPI-PVA > SPI-HPMC > SPI-CNF > SPI > PEO. The only formulation with viscosity like the UF adhesive is the one containing PVA; this might be due to the formation of hydrogen bonds between PVA and protein molecules [76]. Furthermore, the addition of lignin increases the viscosity of all formulations to values very close to the UF adhesive. Interestingly, the formulation with PVA did not increase viscosity upon lignin addition.

**Figure 5:**
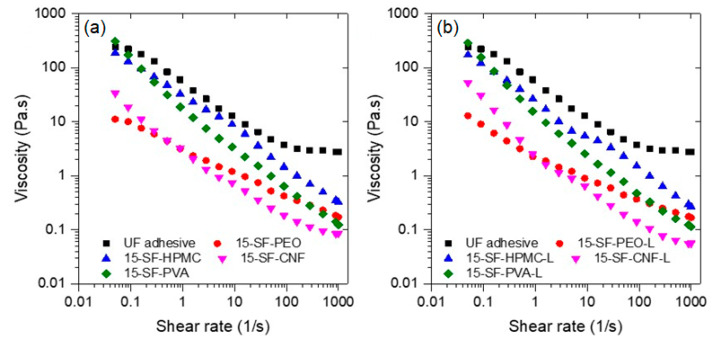
Flow rheology of studied adhesive soy flour formulations (**a**) without and (**b**) with lignin.

Although phase separation occurrence prevented the use of most of the formulations containing soy flour except those containing cellulose nanofibrils (CNF), the viscosity of freshly prepared formulation was measured and is reported in Figure 5. As expected, the formulations exhibit higher viscosity than their counterpart with soy isolate, an effect of the higher soy flour solids content (15 wt%). Addition of lignin to the formulations containing soy flour did not increase the viscosity as in the formulations with soy isolate.

### 3.2. Effect of Type of Wood Species

The dry shear strengths of proposed soy-based adhesives were tested according to standard ASTM D905-08. In addition, a specimen glued using the commercial UF adhesive was tested for further comparison.

In this work, the prepared adhesives were applied to southern yellow pine and white maple as representative hardwood and softwood species. A total of 10 valid testing specimens were used to evaluate the dry shear strength for each type of tested adhesive.

Results of mechanical tests indicate that the dry shear strength at the break point is greatly influenced by wood species (see Table 1 and Table 2). The adhesives applied to southern yellow pine (total mean of dry shear strength of 1161 psi) achieved significantly higher dry shear strength values than on white maple (total mean of dry shear strength of 809 psi). In other words, the difference in mean strength is more than 1.4 times higher for southern yellow pine. This effect was tested on 120 specimens, and the result (P = 0.0000) was determined according to a *t*-test at a 95% significance level.

Furthermore, the adhesive bond failures around 2000 psi (SPI-CNF-L formulation with 9% total solids) occurred at the wood, not at the glue line, indicating that cohesion between adhesive components was better than the strength of the wood itself. Once the cracks were initiated, they continued along the glued surfaces. This crack behavior has been reported in the literature for species with low-density earlywood as well [77]. Thus, the measured shear strength is lower than the true strength of the adhesive.

The oven-dry density of southern yellow pine was calculated to be 478 kg/m^3^ (coefficient of variation 6%), and for white maple it was 721 kg/m^3^ (coefficient of variation 3%). The minimum specific gravity is stated to be 650 kg/m^3^ in standard ASTM D905-08 for the shear strength. Hence, the southern yellow pine did not fulfill the method density requirement, but white maple did, and it showed bond failures in most of the cases.

The moisture content of the southern yellow pine at the time of bonding was 7.3% (coefficient of variation 6%); similarly, white maple had a moisture content of 7.2% (coefficient of variation 3%). Thus, the possible effect of the different moisture content of wood specimens can be disregarded. A sufficient amount of adhesive (0.4 g per 3 in^2^ or 0.1333 g/in^2^) for both wood species was achieved, as some squeeze-out of excess adhesive was visible at the edges. An opposite trend on adhesive bond strength has been reported by Kalapathy [18], where a higher shear strength was achieved for maple test specimens using trypsin-modified soy protein as adhesive.

### 3.3. Effect of Protein Type and Total Solid Content

**Figure 6:**
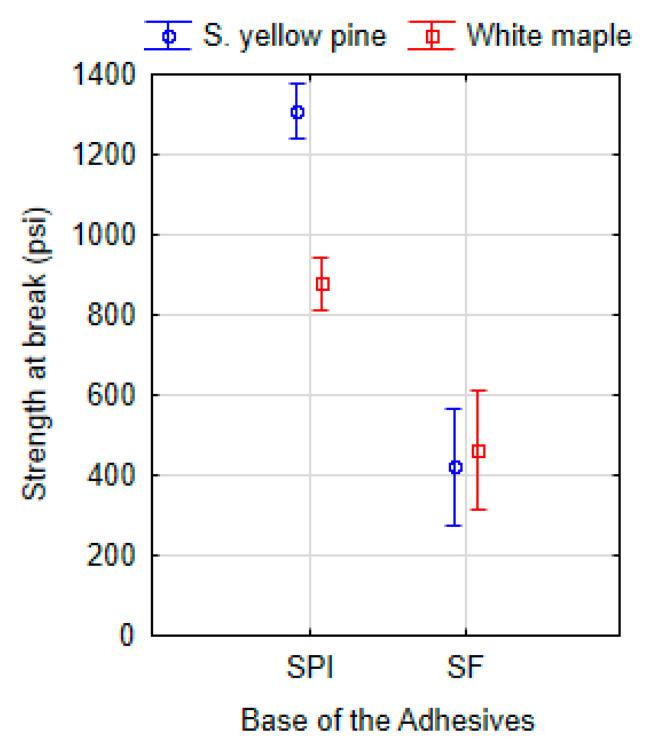
ANOVA (Tukey unequal N HSD) of the dry shear strength at break point according to the base of adhesives: soy protein isolate (10 types: 10-SPI-PEO, 10-SPI-PEO-L, 9-SPI-PEO, 9-SPI-PEO-L, 9-SPI-HPMC, 9-SPI-HPMC-L, 9-SPI-C, 9-SPI-C-L, 9-SPI-PVA, 9-SPI-PVA-L) and soy flour base (2 types: 15-SF-C, 15-SF-C-L).

The ANOVA test (Tukey HSD) showed at 95% confidence level a significant effect between protein types, where the SPI was compared to SF when CNF was used as coadjutant polymer in both cases. The effect was found between adhesives 9-SPI-CNF and 15-SF-CNF, where southern yellow pine (P = 0.000) and white maple (P = 0.003) were used. Similarly, the effect was also found between formulations with lignin addition (9-SPI-CNF-L and 15-SF-CNF-L) for southern yellow pine (P = 0.000) as well as for white maple (P = 0.006). The overall difference in used protein bases of the proposed adhesives is shown in Figure 6. Using ANOVA (Tukey unequal N HSD), a statistical difference is demonstrated between SPI and SF for southern yellow pine (P = 0.000) and white maple (P = 0.001).

The used SF powder had a lower protein content (52%) compared to the commercial SPI (> 90%). The key role in the adhesion can be played by the protein itself as it is considered to be the main contributor of adhesive properties [78]. The difference between SPI and SF resides in the composition and the ratio between content of protein and carbohydrates (soluble and insoluble). Regarding carbohydrates in soy flour, the insoluble ones can cause the strengthening of the adhesive, while soluble carbohydrates are responsible for the increase of viscosity and water absorption [79]. However, all the soluble and insoluble carbohydrates are removed from SPI. The presence of carbohydrates increases with the amount of SF, and carbohydrate content is considered as one factor that causes changes to the adhesive bond strength due to hydroscopic character [10,11,13,80]. This will likely explain why formulations with SPI reached higher shear strength than adhesives with SF.

**Figure 7:**
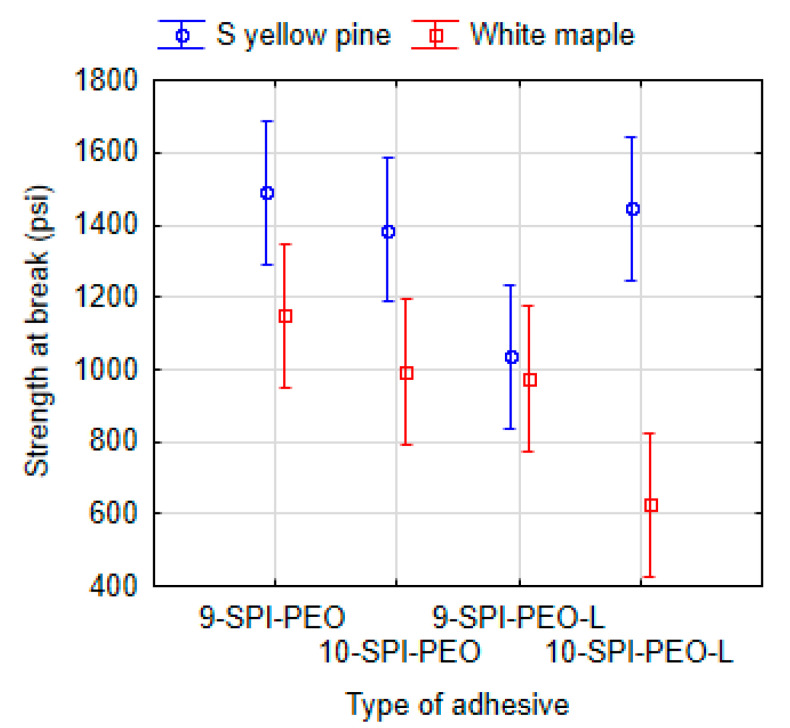
ANOVA of the dry shear strength at the break point and the effect between 9% and 10% total solid on the adhesives with SPI, PEO and lignin variation. Vertical bars denote 95% confidence interval.

The formulations with concentrations of 9% and 10% were compared for the same coadjutant polymer with addition of SPI and PEO and both lignin variation (Figure 7). As expected, there was not a statistically significant effect in 1% difference of total solids in the formulation. The P values for comparison of 9-SPI-PEO with 10-SPI-PEO is P = 0.9999, and P = 0.3070 for comparison of the formulations 9-SPI-PEO-L with 10-SPI-PEO-L, allapplied to white maple. Similarly, no statistical difference was observed between adhesives 9-SPI-PEO and 10-SPI-PEO (P = 1.0000) and 9-SPI-PEO-L and 10-SPI-PEO-L (P = 0.0750) applied to southern yellow pine.

### 3.4. Performance of Soy-Based Adhesives: Effect of Coadjutant Polymer

**Figure 8:**
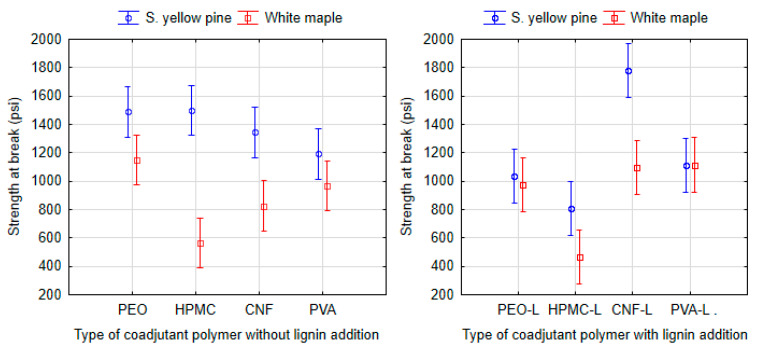
ANOVA of the dry shear strength at break point of proposed adhesives with SPI base and 9% total solid. The left picture shows the dependency of coadjutant type on the shear strength and the right picture shows this dependency with addition of kraft lignin. Vertical bars denote 95% confidence interval.

The shear strength of formulations with different coadjutant polymers (PEO, HPMC, CNF and PVA) was compared only with 9% total solids by using ANOVA (Tukey HSD) at a 95% significance level (Figure 8). No statistically significant differences were found informulations of adhesives without lignin applied to southern yellow pine, and the measured range of average values was from 1193 psi (9-SPI-PVA) to 1488 psi (9-SPI-PEO).

On the other hand, the maximum values of shear strength were achieved on white maple specimens glued using formulations containing PEO (1148 psi) or PVA (967 psi), whereas a significant difference (P > 0.038) was found only when compared to the HPMC formulation (564 psi).

For the adhesive formulations with added lignin applied to white maple, the formulation with HPMC (468 psi) once again proved to have negative effect on the shear strength of adhesives (P > 0.008) compared to adhesives with PEO (973 psi) and PVA (1115 psi) polymers, which exhibit similar strength as the lignin-free formulations. In this case, a statistically significant difference (P = 0.000) was also found for CNF (1098 psi) on white maple. The sample containing CNF (1777 psi), which was applied to the southern yellow pine and in which lignin was added, achieved higher values that were statistically significant when compared to other PEO (1035 psi), HPMC (808 psi) or PVA (1111 psi). This is explained considering that the CNF can act in the proposed adhesives as a nanoscale reinforcement phase, similar to the filler effect reported for composite materials [69,70,72].

### 3.5. Effect of Kraft Lignin Addition

**Figure 9:**
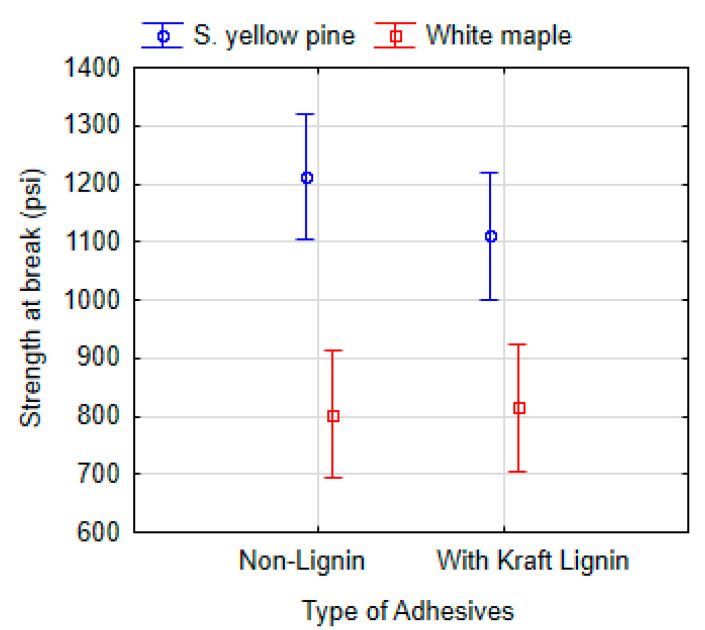
ANOVA of the dry shear strength at the break point according to the non-lignin adhesives (6 types: 10-SPI-PEO, 9-SPI-PEO, 9-SPI-HPMC, 9-SPI-C, 9-SPI-PVA, 15-SF-C) and the adhesives with added kraft lignin (6 types: 10-SPI-PEO-L, 9-SPI-PEO-L, 9-SPI-HPMC-L, 9-SPI-C-L, 9-SPI-PVA-L, 15-SF-C-L). Vertical bars denote 95% confidence interval.

The lignin effect was evaluated by comparing the strengths at the break point of adhesives with added kraft lignin and non-lignin adhesives. The data were analyzed using ANOVA (Tukey HSD) at a 95% significance level (Figure 9). In summary, the adhesives applied to southern yellow pine (P = 0.558) and white maple (P = 0.999) did not prove a statistically significant effect of lignin on strength at break point.

When the adhesives were considered individually applied to southern yellow pine, an increasing of shear strength was found for the formulation containing SPI with 9% total solids and containing CNF and lignin CNF-L when compared to the formulation without lignin (P = 0.0389). The higher values of strength at break observed for SPI-CNF-L formulations compared to SPI-CNF indicate that the presence of lignin enhances the interaction between the adhesive components and southern yellow pine. It has been reported in the literature that it is possible that a crosslinking network is formed due to the reaction between amino acids on the protein molecule with lignin which results in good wet shear strength [25].

Conversely, a negative effect of lignin addition was found in formulations containing SPI with 9% total solid mixed with PEO, HPMC and lignin; PEO-L (P = 0.020) and HPMC-L (P = 0.000), where southern yellow pine was used. These positive and negative effects occurred only for southern yellow pine; no significant effect was observed for the white maple test specimens.

### 3.6. Stability and Bond Line Color of Proposed Protein Adhesives

Regarding stability and shelf life, there was an apparent decrease in viscosity of prepared soy protein adhesive formulations with storage time, although no attempts were made to monitor the change. The viscosity of adhesives significantly affects the shear strength, as at very high viscosity the adhesive is not able to effectively penetrate the substrate [78]. For soybean adhesives, Lambuth [17] mentioned a storage period in a range from 6 to 12 h. In this work, all the mixed non-lignin adhesives stayed in homogeneous liquid form for about 10 days, except the sample 9-SPI-HMPC, which changed to a gel at the end of the fifth day. This was also the only adhesive that created a thick foam during the mixing process. Generally, the addition of kraft lignin increased the viscosity of the soy protein formulations as discussed above; lignin addition also induced gelation on some samples. The same effect was observed by Pradyawong [26], who found that lignin addition slightly increases the thermostability and spreadability of the adhesives.

From the technical standpoint, the color of adhesives should be taken into consideration for some applications, as the proposed protein adhesives with lignin have a dark brown color. After curing, the color of the adhesive mixture was the color of the final glue line. The majority of SF or SPI gave a slightly yellow color to the lignin-free protein adhesives, whereas the addition of kraft lignin produced a glue line with dark red/brown. This dark color may cause an esthetical problem, especially when thin layers of the wood are glued [81]. Otherwise, the strength and functionality of the proposed formulations offer enormous potential as a formaldehyde-free biobased wood adhesive.

### 3.7. Performance of Proposed Adhesives with Relation to UF Adhesive

The mean shear strength of UF adhesive applied to white maple achieved 281 psi, while the value of test specimens with southern yellow pine was 808 psi (see Table 1 and Table 2). The effect of wood species was previously described. When the values of UF were compared to the shear strength of proposed adhesives, it was found that four of the wood formulations achieved significantly better shear strength than commercial UF (P = 0.000) for both wood species. These were the 9% total solids formulation with soy protein isolate and PEO (9-SPI-PEO) and the formulation with 10% total solids (10-SPI-PEO). Similarly, the 9% total solids SPI adhesive formulations containing CNF with lignin (9-SPI-CNF-L) and without lignin addition (9-SPI-CNF) performed better than the UF resin.

## 4. Conclusions

Different wood adhesives containing soy protein isolate and soy protein flour were evaluated. The results of dry shear strength show that the adhesive performance of the formulations is different if they are applied to southern yellow pine or maple. Southern yellow pine specimens achieved significantly higher shear strength (total mean 1161 psi) than white maple test specimens (total mean 809 psi). In addition, the performance of the adhesives formulations varies depending on whether they contain soy isolate or soy flour and also depend on the coadjutant polymer used. Adhesive formulations containing SPI have an enhanced effect on the dry shear strength of glued wood specimens compared to formulations containing SF. The addition of lignin has a different effect depending on the coadjutant polymer in the formulation. A negative effect of lignin addition was observed in formulations containing PEO and HPMC as coadjutant polymers. Conversely, a positive effect of lignin addition was found for SPI adhesives formulation with CNF. The formulation with 9% total solids, SPI-CNF-L, achieved the highest mean strength of all proposed adhesives and this value was 2.2 times higher than the value of UF adhesive (808 psi) when applied to southern yellow pine. Overall, the proposed soy-based wood adhesives meet or exceed the strength of bonding achieved with UF, but without the use of formaldehyde. They represent a sustainable alternative for wood adhesives applications.

## Figures and Tables

**Table 1:** Analyzed data of strength at break of southern yellow pine according to the type of tested adhesive.

	Proposed Adhesives												Commercial Adhesive
Lignin Addition	Non-Lignin						With Lignin						**UF**
Protein Type	SPI					SF	SPI					SF	
Concentration	9%				10%	15%	9%				10%	15%	
Coadjutant Polymer	PEO	HPMC	CNF	PVA	PEO	CNF	PEO	HMPC	CNF	PVA	PEO	CNF	
Mean (psi)	1488 ^a,b^	1501 ^a,b^	1345 ^a,b,c^	1193 ^b,c,d^	1386 ^a,b,c^	359 ^e^	1035 ^c,d^	808 ^d,e^	1777 ^a^	1111 ^b,c,d^	1445 ^a,b,c^	483 ^e^	808 ^d,e^
Mean (MPa)	10.26	10.35	9.27	8.23	9.56	2.48	7.14	5.57	12.25	7.66	9.96	3.33	5.57
Median (psi)	1444	1547	1358	1300	1335	392	1103	707	1758	1088	1479	485	808
Standard Deviation (psi)	408	207	215	261	379	90	493	367	255	281	243	99	296
Minimum (psi)	904	1139	1009	670	641	211	435	377	1481	672	1074	328	299
Maximum (psi)	2130	1756	1789	1487	2048	456	1846	1362	2102	1524	1801	638	1253
Coefficient of Variation (%)	27%	14%	16%	22%	27%	25%	48%	45%	14%	25%	17%	20%	37%

Means with the same letter are not significantly different from each other, according to the HSD test; 145 psi = 1 MPa.

**Table 2:** Analyzed data of strength at break point of white maple according to the type of tested adhesive.

	Proposed Adhesives												Commercial Adhesive
Lignin Addition	Non-Lignin						With Lignin						UF
Protein Type	SPI					SF	SPI					SF	
Concentration	9%				10%	15%	9%				10%	15%	
Coadjutant	PEO	HPMC	CNF	PVA	PEO	CNF	PEO	HMPC	CNF	PVA	PEO	CNF	
Mean (psi)	1148 ^a^	564 ^c,d,e^	826 ^b,c^	967 ^a,b^	995 ^a,b^	318 ^d,e^	973 ^a,b^	468 ^d,e^	1098 ^a,b^	1115 ^a,b^	624 ^c,d^	610 ^c,d^	281 ^e^
Mean (MPa)	7.92	3.89	5.70	6.67	6.86	2.19	6.71	3.23	7.57	7.69	4.30	4.21	1.94
Median (psi)	1185	557	805	858	976	323	895	443	1069	1125	626	546	269
Standard Deviation (psi)	338	154	248	320	124	97	225	98	216	320	133	125	84
Minimum (psi)	654	321	415	507	834	175	744	318	690	399	376	496	180
Maximum (psi)	1881	893	1229	1484	1227	435	1522	679	1459	1494	825	865	466
Coefficient of Variation (%)	29%	27%	30%	33%	12%	30%	23%	21%	20%	29%	21%	20%	30%

Means with the same letter are not significantly different from each other, according to the HSD test; 145 psi = 1 MPa.

## Data Availability

The data presented in this study are available on request from the corresponding author.
